# Residential Proximity to a Major Roadway Is Associated with Features of Asthma Control in Children

**DOI:** 10.1371/journal.pone.0037044

**Published:** 2012-05-17

**Authors:** Meredith S. Brown, Stefanie Ebelt Sarnat, Karen A. DeMuth, Lou Ann S. Brown, Denise R. Whitlock, Shanae W. Brown, Paige E. Tolbert, Anne M. Fitzpatrick

**Affiliations:** 1 Department of Pediatrics, Emory University School of Medicine, Emory University, Atlanta, Georgia, United States of America; 2 Department of Environmental and Occupational Health, Rollins School of Public Health, Emory University, Atlanta, Georgia, United States of America; 3 Children's Healthcare of Atlanta Center for Developmental Lung Biology, Atlanta, Georgia, United States of America; University of Montreal, Canada

## Abstract

**Background:**

While several studies suggest that traffic-related air pollutants are detrimental for respiratory health, few studies have examined relationships between residential proximity to a major roadway and asthma control in children. Furthermore, a major limitation of existing research is reliance on self-reported outcomes. We therefore determined the spatial relationship between the distance from a major roadway and clinical, physiologic and inflammatory features of asthma in a highly characterized sample of asthmatic children 6–17 years of age across a wide range of severities. We hypothesized that a closer residential proximity to a major roadway would be associated with increased respiratory symptoms, altered pulmonary function and a greater magnitude of airway and systemic inflammation.

**Methodology/Principal Findings:**

224 children 6–17 years with confirmed asthma completed questionnaires and underwent spirometry, plethysmography, exhaled nitric oxide determination, exhaled breath condensate collection and venipuncture. Residential distance from a major roadway was determined by mapping the geographic coordinates of the residential address in Geographic Information System software. The distance between the home address and the nearest major roadway was calculated according to the shortest distance between the two points (i.e., “as the crow flies”). Asthmatic children living in closer proximity to a major roadway had an increased frequency of wheezing associated with increased medication requirements and more hospitalizations even after controlling for potential confounders. These children also had increased airway resistance, increased airway inflammation reflected by a lower breath condensate pH, and higher plasma EGF concentrations.

**Conclusions/Significance:**

These findings suggest that closer residential proximity to a major roadway is associated with poorer asthma control in school-age children. Assessment of residential proximity to major roadways may be useful in the clinical evaluation of asthma in children.

## Introduction

Asthma is a complicated disorder associated with variable airway inflammation and airflow limitation in response to specific triggers. Whereas the majority of children with asthma achieve good symptom control with low doses of inhaled corticosteroids **(ICS)** [1], some children have ongoing symptoms despite treatment with high doses of ICS and even oral corticosteroids [2]. These children with severe, ICS-refractory asthma consume a large proportion of healthcare resources and suffer extreme morbidity [3], [4]. While the factors associated with asthma control in children are not understood, previous studies have demonstrated associations between traffic-related air pollutants and the development of asthma symptoms in infants and preschoolers [5]–[8] and the likelihood of current asthma amongst schoolchildren [9]–[12]. More recent studies have also revealed increased respiratory symptoms in asthmatic children residing in close proximity to a major roadway [13], [14] which correlated with the magnitude of vehicle emissions [14].

These studies suggest that the increased burden of traffic-related air pollutants associated with a close residential proximity to a major roadway may be an important factor in asthma pathogenesis in children. However, the degree to which traffic-related pollution contributes to asthma control in children with existing asthma is not clear. Furthermore, the major limitation of existing research is the use of nonspecific measures of residence such as ZIP codes, self-reported traffic density, and self-reported health outcomes. Because studies utilizing objective measures of asthma have been few and limited, we sought to examine the spatial relationship between distance from a major roadway and asthma features in a highly characterized sample of children across a wide spectrum of asthma severity. We hypothesized that a closer residential proximity to a major roadway would be associated with an increased burden of wheezing as well as increased medication use and healthcare utilization, altered pulmonary function, and increased markers of airway and systemic inflammation.

**Figure 1:**
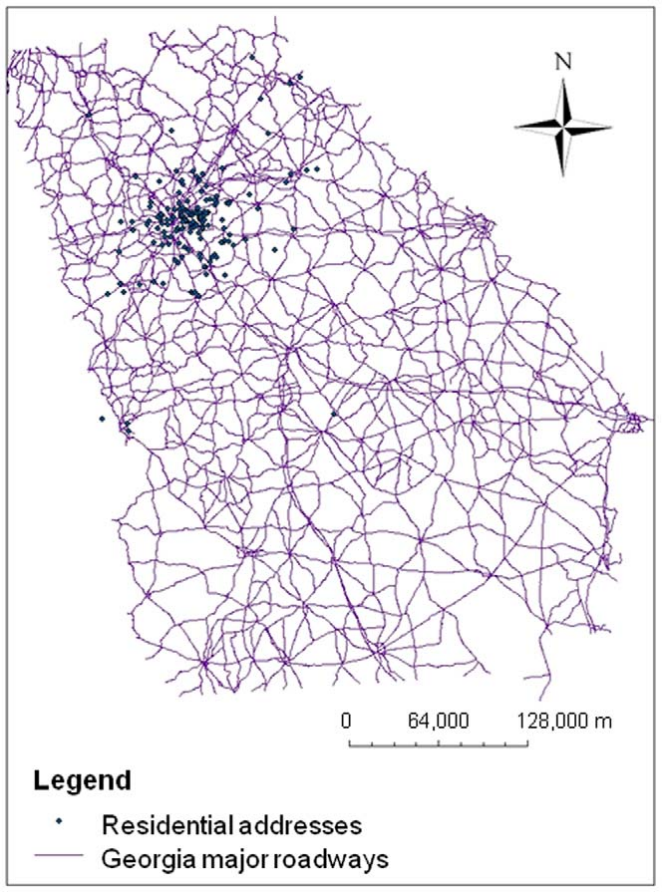
Residential addresses mapped with an overlay of major roadways in Georgia.

## Methods

A convenience sample of children 6–17 years of age with physician-diagnosed asthma was selected from a pediatric asthma clinic in Atlanta, Georgia. Although located within metropolitan Atlanta, this clinic serves a racially and socioeconomically diverse population of children across a 20-county region. All children had historical evidence of ≥12% reversibility in the forced expiratory volume in one second **(FEV_1_)** after short-acting bronchodilator administration and had been under the care of an asthma subspecialist for at least 12 months. Other inclusion criteria included a stable residence for the past 12 months and the ability to speak and understand English. Exclusion criteria included premature birth before 34 weeks gestation or other co-morbid pulmonary disorders, such as immunodeficiency or aspiration disorders. Sinus disease and gastroesophageal reflux were not criteria for exclusion provided they were appropriately treated and controlled for at least 12 months.

Children were invited to participate in the study by the Principal Investigator (AMF). Children and their caregivers presenting for routine clinical care were informed of the study during their clinic appointment and were asked if they would like to receive a telephone call from the lead study coordinator (DRW) to learn more about the study details. Children willing to participate in the study were scheduled for a research-only visit.

**Table 1:** Features of the subjects according to residential proximity from a major roadway.

	All subjects	<417 meters	>417 meters	p-value
	(n = 224)	(n = 56)	(n = 168)	
Age (years)	12±4	12±5	12±3	0.991
Male	123 (55)	31 (55)	92 (55)	1
Non-white	153 (68)	44 (79)	109 (65)	0.068
Duration of asthma (years)	9±4	9±5	9±4	0.691
Parent with current asthma	110 (49)	32 (57)	78 (46)	0.17
Daily asthma controller medications				
ICS (single agent)	48 (21)	9 (16)	39 (23)	0.347
ICS+long-acting beta-agonist	151 (67)	44 (79)	107 (63)	0.048
Montelukast	173 (77)	50 (89)	123 (73)	0.016
Systemic corticosteroids	18 (8)	7 (13)	11 (7)	0.164
Environmental tobacco smoke exposure (≥1 day per week)	45 (21)	15 (28)	30 (18)	0.173
Allergic rhinitis	204 (91)	50 (89)	154 (92)	0.593
Atopic dermatitis	125 (56)	36 (64)	89 (53)	0.163
Blood eosinophils (%)^1^	5±5	5±5	5±4	0.736
Serum IgE (kU/L)^1^	525±822	490±645	536±874	0.328
Co-morbid conditions				
Obesity (BMI>95^th^ percentile)	52 (23)	16 (29)	36 (22)	0.518
Previous pneumonia	126 (57)	30 (55)	96 (58)	0.754
Recurrent sinusitis	99 (45)	26 (47)	73 (44)	0.755
Gastroesophageal reflux	63 (29)	22 (40)	41 (25)	0.038
Private health insurance	150 (67)	31 (55)	119 (71)	0.048

Data represent the mean ± SD or the frequency (%).

1 Data were logarithmically transformed prior to statistical analysis.

### Ethics statement

This study was conducted under approval from the Emory University Institutional Review Board. Written informed consent at the research-only visit was obtained from the parents or legal guardians of participating children. Children 12–17 years also provided written assent while children 6–11 years provided verbal assent to the study procedures.

### Characterization procedures

Children were assessed during a research-only outpatient visit. The investigators and research coordinators responsible for subject assessment and measurement procedures were blinded to the address and residential proximity to a major roadway during the study visit. The study visit was rescheduled if any of the following were reported within the preceding 4-week period: 1) upper respiratory viral symptoms such as rhinorrhea, 3) acute worsening of asthma symptoms, 3) antibiotic use or 4) systemic corticosteroid use. Children whose lung function was not within 10% of their baseline (assessed during the most recent clinical visit while well) were also rescheduled. Participating children and their caregivers completed questionnaires about asthma symptoms over the past 4 weeks and healthcare utilization over the preceding 12 months. Self-reported asthma characteristics and medical history information including asthma medications, co-morbid conditions and healthcare utilization were verified by a review of medical records.

Spirometry was performed in the presence of daily medication with a portable spirometer (KoKo® PDS, Ferraris, Louisville, CO). Subjects were asked to withhold short-acting bronchodilators and caffeine for at least 4 hours prior to the procedure. Results met criteria for reproducibility [15] and the best of three forced vital capacity **(FVC)** maneuvers was interpreted. Population reference equations were used to calculate percent predicted values for FEV_1_ and the mid-expiratory flow rate at 25–75% of vital capacity **(FEF_25–75_)** [16]. Total lung capacity **(TLC)**, residual volume **(RV)** and airway resistance were measured with a body plethysmograph (MedGraphics® Elite Series,™ St. Paul, MN) and expressed according to reference standards [17]. Exhaled nitric oxide concentrations were analyzed using online methods (NIOX MINO®, Aerocrine Inc., New Providence, NJ) with a fixed flow rate of 0.05 L/second and exhalation duration of 6 seconds [18].

Breath condensate was collected for 15 minutes with a polypropylene breath collection device with a one-way inhalation valve and condensation tube chilled at −80°C (R-tube®, Respiratory Research, Inc., Charlottesville, VA). Samples were deaerated with argon for 8 minutes and pH was determined before and after deaeration with a pH meter and probe (Orion 525™ and Orion 98-03,™ Thermo Electron Corporation, Beverly, MA).

Whole blood and serum were analyzed for the percentage of eosinophils and serum immunoglobulin E **(IgE)** concentrations, respectively, by a commercial laboratory (Quest Diagnostics, Tucker, GA). Epidermal growth factor **(EGF)**, vascular endothelial growth factor **(VEGF)**, tumor necrosis factor alpha **(TNFα)**, interferon gamma **(IFNγ)**, IL-4, IL-5, IL-6, IL-8, IL-10 and IL-13 were measured in the plasma with a commercial bead-based assay with a sensitivity of 0.1 to 5.7 pg/mL (Millipore, Billerica, MA). Data were analyzed using the BioRad Bio-Plex® System (Bio-Rad Laboratories, Hercules, CA) with gates of 4335 and 10,000.

### Residential geocoding approaches

The residential distance from a major roadway was determined for each subject by mapping the mapping the geographic coordinates of the subject's residential address in Geographic Information System software (ArcGIS, Version 10, Esri, Redlands, CA). The ArcGIS geocoder function was used to determine the exact geographic coordinate based on the residential street and house number. Addresses that were not matched by ArcGIS (n = 27) were matched using Google Earth (Version 6.0, Google, Mountain View, CA) to obtain X and Y coordinates that were then entered into the ArcGIS software. A subset of ten address records that were geocoded using ArcGIS were then geocoded using GoogleEarth to validate that identical results were obtained from ArcGIS and GoogleEarth. The map containing all subjects' residential locations was then overlaid with the major roadways of Georgia. The major roadways were obtained from the 2010 National Transportation Atlas Database from The Bureau of Transportation Statistics [19]. This analysis considered major roadways to be all state highways and interstates as dictated by the United States Department of Transportation. Using the ArcGIS measuring tool, the distance between each subject's home and the closest major roadway [e.g. distances between all of the points (addresses) to the nearest line segment (roadways)] was calculated in meters. The distance between the home address and the nearest major roadway was calculated according to the shortest distance between the two points (i.e., “as the crow flies”).

### Outcome variables

The primary outcome variable was poor asthma symptom control, defined as wheezing episodes requiring short-acting bronchodilator use that occurred more than twice weekly (averaged over a period of 4 weeks). The primary outcome was assessed by child and caregiver self-report. Although other respiratory symptoms such as cough and dyspnea are components of asthma control [1], these symptoms were not included as part of the primary outcome assessment due to their potential association with other co-morbid conditions including allergic rhinitis, sinusitis, obesity, and vocal cord dysfunction. Secondary outcomes included healthcare utilization, pulmonary function indices and inflammatory biomarkers.

### Statistical analyses

Data were analyzed with PASW® software (Version 19, SPSS Inc., Chicago, IL) after logarithmic transformation of variables that were not normally distributed. T-tests and chi-square tests were used for group differences and logistic regression was used to determine the association between residential distance from a major roadway and measures of asthma severity. Potential confounders and covariates were controlled for in the models. Significance was defined as α<0.05 using two-tailed tests.

## Results

Two hundred and fifty children were initially approached for the study. Two hundred twenty four children agreed to learn more about the study and were subsequently enrolled. Demographic variables such as geographic region, sex, age, and asthma severity did not differ between children who were and were not enrolled. Of the 224 enrolled subjects, 5 children were rescheduled for acute worsening of asthma symptoms (presumably due to viral illnesses) that required systemic corticosteroid treatment. These 5 children were distributed randomly across metropolitan Atlanta and only their follow-up data when well were included in the analysis.

The majority of children enrolled in the study resided within metropolitan Atlanta (**Figure 1**). The distance between each subject's home and the closest major roadway ranged from 0.23 to 9633 meters (25^th^ percentile: 417 meters, 50^th^ percentile: 1074 meters, 75^th^ percentile: 2208 meters) and was comparable to the average distance within the larger metropolitan Atlanta area. To determine the optimum cut-point for analyses, quartiles of roadway distance were first evaluated in relation to the primary outcome, with the furthest quartile as the reference group. Children in the first quartile who resided within 417 meters of a major roadway had increased odds of wheezing more than twice weekly (unadjusted OR 2.64, 95% CI: 1.19–5.83). No significant associations between roadway proximity and wheezing were observed for the other quartiles so resulting analyses were performed using a cut-point of 417 meters. Because other studies suggest that the concentration of emitted motor vehicle pollutants may be highest within 150 meters of a roadway [14], exploratory analyses were also conducted with a cut-point of 150 meters.

Features of participating children are shown in **Table 1**. Although age and sex did not differ, children who resided within 417 meters of a major roadway were treated with more asthma controller medications aside from ICS and had a higher frequency of physician-diagnosed gastroesophageal reflux. Children living within 417 meters were also less likely to have private health insurance. To minimize or control potential sources of confounding of the primary and secondary outcomes, the resulting models were adjusted for payor status (private insurance versus Medicaid) as a proxy of socioeconomic status, race (white versus non-white), parental history of asthma, environmental tobacco smoke exposure at least one day per week, and history of gastroesophageal reflux.

### Wheezing frequency

The primary outcome was wheezing requiring short-acting bronchodilator use more than two days per week. Children residing within 417 meters of a major roadway had increased odds of wheezing more than two days per week compared to children residing further than 417 meters (**Table 2**). The odds of daily wheezing, defined as wheezing requiring short-acting bronchodilator use at least 5 of 7 days per week, were also increased in this group (**Table 2**). Similar findings were observed in exploratory analyses using a cut-point of 150 meters (**Table 3**).

**Table 2:** Associations between residential proximity to a major roadway (<417 meters [N = 56] versus >417 meters [N = 168]) and primary and secondary outcomes in asthmatic children.

Outcome variable	Children living <417 meters with outcome	Children living >417 meters with outcome	Adjusted	95% CI	p-value
	N (%)	N (%)	OR^1^		
Wheezing more than twice weekly	27 (48)	43 (26)	2.24	1.14, 4.42	0.02
Daily wheezing (5 of 7 days per week)	13 (23)	16 (9)	2.63	1.10, 6.26	0.029
Healthcare utilization for asthma (previous 12 months)					
Emergency room visit	40 (71)	86 (51)	1.86	0.92, 3.76	0.086
Hospitalization^2^	31(55)	48 (29)	2.45	1.23, 4.89	0.011
Intensive care unit admission	19 (34)	29 (17)	2.41	1.08, 5.36	0.031
Airflow limitation					
FEV_1_<80% of predicted value	16 (29)	35 (21)	1.27	0.59, 2.71	0.544
FEV_1_/FVC<80% of predicted value	15 (27)	26 (15)	2.03	0.93, 4.44	0.075
FEF_25–75_<80% of predicted value	39 (70)	89 (53)	2.26	1.04, 4.95	0.041

1 Adjusted for payor status (private insurance versus Medicaid), race (white versus non-white), parental history of asthma (yes versus no), environmental tobacco smoke exposure at least one day per week (yes versus no), and history of gastroesophageal reflux (yes versus no).

2 Defined as a hospital stay ≥24 hours.

**Table 3:** Associations between residential proximity to a major roadway (<150 meters [N = 18] versus >150 meters [N = 206]) and outcomes in asthmatic children identified through exploratory analyses.

Outcome variable	Children living <150 meters with outcome	Children living >150 meters with outcome	Adjusted	95% CI	p-value
	N (%)	N (%)	OR^1^		
Wheezing more than twice weekly	13 (72)	57 (28)	6.05	1.87, 19.56	0.003
Daily wheezing					
(5 of 7 days per week)	7 (39)	22 (11)	3.93	1.24, 12.45	0.02
Healthcare utilization for asthma (previous 12 months)					
Emergency room visit	11 (61)	115 (56)	1.04	0.34, 3.18	0.945
Hospitalization^2^	11 (61)	68 (33)	3.18	1.01, 9.94	0.047
Intensive care unit admission	6 (33)	42 (20)	1.63	0.46, 5.74	0.445
Airflow limitation					
FEV_1_<80% of predicted value	5 (28)	46 (22)	1.32	0.41, 4.24	0.636
FEV_1_/FVC<80% of predicted value	5 (28)	36 (17)	1.71	0.52, 5.64	0.376
FEF_25–75_<80% of predicted value	13 (72)	114 (55)	3.44	0.87, 13.57	0.078

1 Adjusted for payor status (private insurance versus Medicaid), race (white versus non-white), parental history of asthma (yes versus no), environmental tobacco smoke exposure at least one day per week (yes versus no), and history of gastroesophageal reflux (yes versus no).

### Medication requirements and healthcare utilization

Consistent with the increased frequency of wheezing, children residing within 417 meters of a major roadway were prescribed a higher cumulative daily dose of ICS (mean ± SD, 685±344 vs. 564±374 mcg of fluticasone equivalent per day, p = 0.033) and more asthma controller medications aside from ICS for symptom control (two or more additional controller medications, 77% vs. 55%, p = 0.004).

Medications requirements were not higher in children residing within 150 meters of a major roadway.

Emergency room visits for asthma within the preceding 12 months did not differ in children residing within 417 meters of a major roadway. However, children living less than 417 meters from a major roadway had increased odds of hospitalization for asthma within the preceding 12 months, as well as increased odds of intensive care unit admissions (**Table 2**). A similar trend for hospitalization was also noted for children residing within 150 meters of a major roadway (**Table 3**).

### Pulmonary function

**Figure 2:**
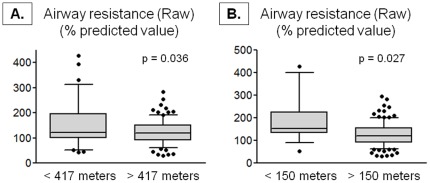
Airway resistance (Raw) percent predicted values in children with a residential proximity (A) within 417 meters (<417 meters, n = 37; >417 meters, n = 96) and (B) within 150 meters (<150 meters, n = 17; >150 meters, n = 116) of a major roadway. Whiskers represent the 10^th^ to 90^th^ percentile and dots represent individual data points. Significance testing was adjusted for payor status, race, parental history of asthma, environmental tobacco smoke exposure, and gastroesophageal reflux.

FVC, FEV_1_, and FEF_25–75_ values were not significantly different in children living within 417 meters from a major roadway (<417 meters vs. >417 meters, FVC: 101±16 vs. 101±16%, p = 0.928; FEV_1_: 88±20 vs. 92±18%, p = 0.143; FEF_25–75_: 68±38 vs. 79±37%, p = 0.091). However airflow limitation assessed by FEV1/FVC was greater in children residing within 417 meters in univariate analyses (absolute ratio of FEV_1_/FVC: 0.75±0.11 vs. 0.79±0.10, p = 0.022; % predicted value, 87±13 vs. 90±11, p = 0.029). After adjustment for potential confounders, the only significant finding was airflow limitation assessed by an FEF_25–75_ value less than 80% of predicted norms [16] (**Table 2**). While lung volume measurements also did not differ between groups (mean ± SD, % predicted value, <417 meters vs. >417 meters, TLC: 100±15 vs. 100±17%, p = 0.998; RV: 143±69 vs. 132±68, p = 0.473; RV/TLC: 0.30±0.12 vs. 0.28±0.12, p = 0.353), children living within 417 meters from a major roadway had higher airway resistance (**Figure 2A**). Airway resistance was similarly increased in children residing within 150 meters of a major roadway (**Figure 2B**).

### Markers of inflammation

**Figure 3:**
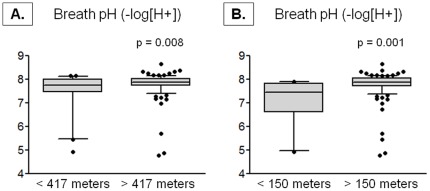
Deaerated breath pH in children with a residential proximity (A) within 417 meters (<417 meters, n = 25; >417 meters, n = 99) and (B) within 150 meters (<150 meters, n = 10; >150 meters, n = 114) of a major roadway. Whiskers represent the 10^th^ to 90^th^ percentile and dots represent individual data points. Significance testing was adjusted for payor status, race, parental history of asthma, environmental tobacco smoke exposure, and gastroesophageal reflux.

**Figure 4:**
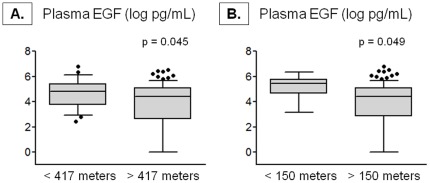
Plasma epidermal growth factor (EGF) concentrations in children with a residential proximity (A) within 417 meters (<417 meters, n = 26; >417 meters, n = 91) and (B) within 150 meters (<150 meters, n = 10; >150 meters, n = 108) of a major roadway. Whiskers represent the 10^th^ to 90^th^ percentile and dots represent individual data points. Significance testing was adjusted for payor status, race, parental history of asthma, environmental tobacco smoke exposure, and gastroesophageal reflux.

Exhaled nitric oxide concentrations did not differ in children residing within 417 meters of a major roadway (mean ± SD, <417 meters vs. >417 meters, 37±34 vs. 34±35 ppb, p = 0.607). However, exhaled breath condensate deaerated pH was significantly lower in children residing with 417 meters of a major roadway (**Figure 3A**). Although plasma concentrations of VEGF, TNFα, IFNγ, IL-4, IL-5, IL-6, IL-8, IL-10, and IL-13 were not associated with residential proximity to a major roadway and failed to distinguish the proximity groups (data not shown), plasma EGF concentrations were significantly higher in children living within 417 meters of a major roadway (**Figure 4A**). Similar differences in exhaled breath condensate deaerated pH and plasma EGF were observed in children residing within 150 meters of a major roadway (**Figure 3B** **,** **Figure 4B**).

## Discussion

To our knowledge, this is the first study to assess clinical, physiologic, and inflammatory features of asthma and their relationships to roadway proximity in a highly-characterized sample of children with asthma in the United States. A unique strength of the study is the use of objective geographic identifiers and asthma characteristics and outcomes, as opposed to pure self-report, which may be significantly biased [20]. Another strength is the inclusion of only asthmatic children, since asthma medications and other key variables in the medical history may confound the spatial relationship between roadway proximity and asthma control [21], [22]. Here, we found that asthmatic children with a residential proximity closer to a major roadway (i.e., within 417 meters) had more frequent asthma-related wheezing episodes. Perhaps as a function of increased symptoms, these children were prescribed higher doses of ICS and more asthma controller medications but had increased hospitalizations for asthma, increased mid-expiratory airflow limitation and airway resistance. Although exhaled nitric oxide concentrations were not increased as a function of roadway proximity, children who lived closer to a major roadway also had increased generalized airway and systemic inflammation reflected by lower breath condensate deaerated pH values and higher plasma concentrations of EGF, which is associated with airway tissue remodeling in children [23]. While we failed to consistently confirm all of these observations in children living within 150 meters of a major roadway, this is likely due to the small sample size of children within this group (n = 18) which resulted in decreased power for these exploratory analyses. Regardless, these data support the hypothesis that a closer residential proximity to a major roadway is associated with features of asthma severity in children. These findings further suggest that the spatial relationship between the distance from a major roadway and asthma features may be useful in the clinical assessment of asthma severity in this age group.

Atlanta, Georgia, has a disproportionate prevalence of asthma and affected children have an increased burden of respiratory symptoms as compared to other geographic areas [24]–[26]. Atlanta is also plagued by some of the highest numbers of smog alert days in the United States, as well as heavy amounts of traffic [27]. During the Olympic Games hosted in Atlanta in 1996, efforts to reduce downtown traffic congestion resulted in a 28% reduction in peak daily ozone concentrations and an 11–44% reduction in acute asthma events that were directly attributable to decreased traffic density and vehicle emissions in the area [28]. While vehicle emissions in Atlanta may be less severe as compared to other international locations, traffic-related air pollutants are a composite mixture of nitrogen oxides, particulate matter, carbon monoxide, sulfur oxides and other volatile organic compounds which all function as airway irritants in children [29]. Indeed, recent studies in the United States [30]–[32] and Mexico [14], [33] have demonstrated associations between vehicle-related emissions and respiratory symptoms (namely wheezing) among asthmatic children. Other studies have similarly shown greater airflow limitation [14], [34], [35] and an increased risk of acute exacerbations [36], [37] and repeated hospitalizations for asthma [38] among children with high vehicle emission exposures. While these observations warrant further study and validation in other subpopulations of asthmatic children, these findings suggest that traffic-related air pollutants may be an important modulator of asthma control in children.

Although previous studies of general populations of children have demonstrated associations between exhaled nitric oxide concentrations and air pollution exposure [39], [40], we did not detect significant relationships between residential roadway proximity and exhaled nitric oxide in this study. However, this is likely due to the unique nature of our sample, since 85% of enrolled children had persistent symptoms necessitating daily ICS therapy. Whereas several studies have demonstrated the utility of exhaled nitric oxide in the diagnosis of asthma and assessment of asthma control [41], others have shown consistent reductions in exhaled nitric oxide concentrations with ICS therapy [42]. The utility of exhaled nitric oxide monitoring is further reduced in subjects treated with high doses of ICS [43]. Thus while Holguin et al. [14] and others [44], [45] have shown associations between air pollution exposure and asthma control in children, the vast majority (i.e., 70–100%) of subjects in those studies were ICS-naïve. The usefulness of exhaled nitric monitoring in subjects with persistent asthma requiring daily ICS therapy is therefore less clear.

Despite negative associations between roadway proximity and exhaled nitric oxide in this study, we did observe significant associations between residential proximity to a major roadway and other inflammatory markers, including exhaled breath condensate pH and the inflammatory cytokine, EGF. Although breath condensate pH is considered to be an “emerging” biomarker in asthma [46], other studies have shown that breath pH values are lower in children with asthma [47] compared to healthy control populations [48], and these low values are thought to reflect a biochemical disturbance in the airway epithelium associated with airway inflammation [49], [50]. Furthermore, other studies have similarly shown reductions in breath pH with air pollution exposure [51], [52], similar to what was observed here. Although we failed to simultaneously detect significant differences in a number of systemic pro-inflammatory cytokines, it is possible that air pollution results in localized (i.e., airway) versus systemic patterns of inflammation in exposed subjects. However, we did observe higher plasma concentrations of EGF in children living closest to a major roadway. Because EGF is also associated with fibrocyte activation and airway remodeling [53], this finding may account for the increase in small airways airflow limitation observed in this study, although other studies of air pollution and cytokine expression are clearly warranted.

An interesting but unexpected finding in the present study was the association between residential proximity to a major roadway and physician-diagnosed gastroesophageal reflux. Although the mechanisms linking gastroesophageal reflux and asthma are unclear, several studies have demonstrated a higher prevalence of gastroesophageal reflux in subjects with asthma as compared to controls [54], [55], which is further associated with lower breath condensate pH values in this population [50]. Subjects with proximal gastroesophageal reflux as reflected by 24-hour esophageal pH probe monitoring are also more likely to experience greater asthma and health-related quality of life [56]. Thus while recent clinical trials of proton pump inhibitors for gastroesophageal reflux for subjects with asthma have failed to result in clinically significant changes in asthma control [57], [58], gastroesophageal reflux remains a common co-morbid condition in subjects with asthma and is therefore frequently controlled for in other asthma association studies [59]. This study does have limitations. Although we did not include a specific measure of traffic density, traffic density was secondarily controlled by the use of the U.S. Department of Transportation definition of a major highway or interstate, which, by design, is a roadway with a high traffic volume. While we also did not directly measure traffic-related air pollutant concentrations near the residence of each subject, other studies utilizing proximal distance to a major roadway have shown the measure to be a useful approximation of traffic-related air pollutant exposures that may reflect variability in traffic exposures across the population [13], [38], [60], [61]. Our findings of increased generalized airway and systemic inflammation reflected by lower breath condensate pH and increased plasma EGF concentrations in children closer to a major roadway are also in keeping with other reports of increased traffic-related inflammation in these subjects [52], [62]. However, it is important to note that school bus exposures, modes of transportation and the location of each child's school were not recorded in this study, and thus participating children may have had higher or lower traffic exposures than what was reported here. Similarly, although all subjects included in this study had a stable residence for one year and assessment of healthcare utilization was not extended beyond the preceding 12 months, the lifetime residential history of each subject was not available for analysis and may limit interpretation of our results. Other significant sources of confounding may include other un-measured environmental factors and socio-cultural factors, such as family income and education level. Furthermore, a selection bias cannot be ruled out since this sample of children was selected from an academic medical center in metropolitan Atlanta and therefore may be more likely to be non-white, of lower socioeconomic status, and live in multi-dwelling housing. However, this medical center does serve a racially and socioeconomically diverse population of children across a 20-county region. The average distance between each subject's home and the closest major roadway also did not follow a clear systematic pattern and was similar to the metropolitan Atlanta population at large [19].

In conclusion, we have shown that children residing closer to a major roadway have an increased frequency of wheezing associated with increased medication requirements, greater healthcare utilization, increased airflow limitation and airway resistance, and increased inflammation. These findings suggest an important spatial relationship between the distance from a major roadway and asthma control in children. Future studies involving objective markers of traffic density and ambient exposure assessments may help to inform the underlying pollutant components and the mechanisms involved. Given the small but relevant number of attributable cases of respiratory disorders due to vehicle-related pollutants [63], [64], strategies to reduce traffic exposure may be warranted in children with asthma, particularly in children with asthma that is difficult to control.
